# Phloroglucinol Strengthens the Antioxidant Barrier and Reduces Oxidative/Nitrosative Stress in Nonalcoholic Fatty Liver Disease (NAFLD)

**DOI:** 10.1155/2021/8872702

**Published:** 2021-01-14

**Authors:** Krzysztof Drygalski, Katarzyna Siewko, Andrzej Chomentowski, Cezary Odrzygóźdź, Anna Zalewska, Adam Krętowski, Mateusz Maciejczyk

**Affiliations:** ^1^Clinical Research Center, Medical University of Bialystok, Poland; ^2^Department of Endocrinology, Diabetology and Internal Medicine, Medical University of Bialystok, Poland; ^3^Department of Biophysics, Medical University of Bialystok, Poland; ^4^Department of Molecular and Systems Biology, Institute of Bioorganic Chemistry, Polish Academy of Sciences, Poznań, Poland; ^5^Experimental Dentistry Laboratory, Medical University of Bialystok, Poland; ^6^Department of Hygiene, Epidemiology and Ergonomics, Medical University of Bialystok, Poland

## Abstract

Nonalcoholic fatty liver disease (NAFLD) is one of the most commonly occurring diseases within western dietary patterns. Usually untreated, it may lead to type 2 diabetes mellitus (T2DM), steatohepatitis (NASH), and hepatocellular carcinoma (HCC). Besides its severe aftermath, up to now, there is no known therapeutic approach to this disease in everyday clinical practice. Most NAFLD patients are encouraged to do physical activities or diet change and remain without pharmacological treatment. In this study, we present phloroglucinol (PHG) as a novel and promising compound in NAFLD treatment. PHG significantly increased the level of enzymatic and nonenzymatic antioxidants both in palmitate and hydrogen peroxide-induced oxidative stress models. Strengthened antioxidative defense reduced the oxidative/nitrosative damage to cell proteins, lipids, and carbohydrates. Furthermore, PHG treatment reduced hepatic steatosis; lowered inflammatory markers, such as NF-*κ*B or HIF-1*α*; and inhibited cell apoptosis. Moreover, PHG had a more comprehensive effect than other commonly used antioxidants: N-acetylcysteine (NAC) and *α*-lipoic acid (ALA), suggesting its clinical usability. Therefore, our paper supports the benefits of natural compounds as a therapeutical approach to NAFLD.

## 1. Introduction

Nonalcoholic fatty liver disease (NAFLD) is a chronic medical condition associated with the excessive accumulation of free fatty acids, diglycerides, and triglycerides in the liver [1]. NAFLD's leading cause has been attributed to excessive high fat intake referred to as “western dietary pattern.” It is estimated that around 25% of the world's population might be affected, whereas, in western countries, prevalence is higher [2]. The lipid overload state present in NAFLD results in a dysregulation of hepatocytes' metabolic activity, leading to inflammatory response and liver tissue injury [3]. The most common aftermath of liver steatosis in NAFLD is insulin resistance, leading to type 2 diabetes mellitus (T2DM) [4–6]. Additionally, patients affected by NAFLD are also more likely to develop systemic hypertension, while frequent vascular complications increase the rate of cardiac incidents [7–9]. What is more, long-lasting steatosis induces oxidative stress and inflammation which results in the development of nonalcoholic steatohepatitis (NASH). It remains the leading cause of hepatocellular carcinoma (HCC) in western countries [1–3].

Oxidative stress plays a critical role in the progression of NAFLD [10, 11]. Generally, oxidative stress refers to the imbalance between the formation of oxygen/nitrogen free radicals and the efficiency of mechanisms responsible for their elimination. Under physiological conditions, reactive oxygen species (ROS) formed in metabolic processes are effectively scavenged by enzymatic and nonenzymatic antioxidants. However, under pathology, redox imbalance occurs in favor of oxidation reactions, and as a consequence, overproduction of ROS causes oxidative damage to biomolecules and cell structures [12]. The oxidative damage may manifest as both protein and lipid oxidation and the creation of advanced glycation end products (AGEs). Increased production of ROS in NAFLD relates to blockage of *β*-oxidation, which triggers an impairment of the mitochondrial electron transport chain [13]. What is more, the incomplete oxidation of acyl-carnitine causes aggregation of lipotoxic intermediates, which can be an indirect source of ROS [14]. Additionally, recent studies showed the possible role of cytochrome CYP2E1 as a source of ROS in the liver tissue [15]. Since there is no approved medication for NAFLD, whereas mild to moderate lifestyle changes do not bring significant benefits to NAFLD patients, it is crucial to investigate potentially effective drug treatment [16]. Current therapeutic approaches focus their efforts on the reduction of oxidative stress. As for now, silymarin, vitamins E and D, polyunsaturated fatty acids of the omega-3 series, coenzyme Q10, berberine, and curcumin are considered to exert moderate effects after prolonged use [17]. Out of the presented substances, the effects of vitamin E seem to be best documented. However, there are some concerns about the safety of vitamin E supplementation. It has been reported that oral vitamin E supplementation among healthy men increases prostate cancer risk and risk for hemorrhagic stroke in the general population and heart failure in patients with left ventricular dysfunction [18–20]. Based on the above information, we chose phloroglucinol (PHG), a phenolic compound of natural origin, mainly known for its nonspecific antispasmodic properties in gastric tract disorders. Phloroglucinol is safe to use and constitutes an active ingredient in various antispasmodic compositions [21]. Indeed, it has been proven that PHG may have anti-inflammatory and antioxidant capabilities in different medical conditions [22, 23]. Some studies even report its anticancer potential [24, 25]. Nevertheless, it has never been assessed as a potential drug counteracting NAFLD and preventing its progress towards NASH.

In our study, we developed a HepG2 cell line to assess their response to the changes in oxidative balance caused by NAFLD and the feasibility of alleviating these effects using PHG. Furthermore, we compared the effectiveness of PHG with two other compounds of renowned antioxidative properties: N-acetylcysteine (NAC) and *α*-lipoic acid (ALA). The oxidative stress was generated in two models: hydrogen peroxide or palmitic acid-induced steatosis.

## 2. Results

### 2.1. Cell Viability

**Figure 1:**
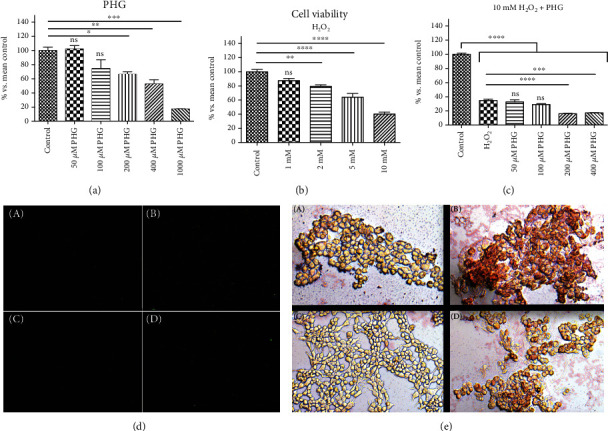
The effect of PHG on cell viability and steatosis. (a, c) PHG was nontoxic, both referring to the control and H_2_O_2_ in concentrations below 200 *μ*M and dose-dependently lowered cell viability in concentrations above 200 *μ*M. (b) H_2_O_2_ showed a dose-dependent effect on cell viability with GI_50_ around 10 mM. (d) Immunofluorescence staining of active Caspase 3 (A: control group; B: NAFLD; C: PHG; D: NAFLD+PHG). (e) Oil Red O staining of HepG2 cells (A: control group; B: NAFLD; C: PHG; D: NAFLD+PHG).

PHG showed a dose-dependent decrease of cell viability which was statistically significant in concentrations above 100 *μ*M (200 *μ*M: -33.6% *p* < 0.05; 400 *μ*M: -47.3% *p* < 0.01; 1000 *μ*M: -82.7% *p* < 0.001). H_2_O_2_ also decreased cell viability in a dose-dependent manner, which was significant in concentrations above 1 mM (2 mM: -20.4% *p* < 0.01; 5 mM: -35.8% *p* < 0.0001; 10 mM: -59.7% *p* < 0.0001). With the addition of PHG to 10 mM H_2_O_2_, the medium did not affect cell viability in concentrations below 200 *μ*M of PHG. Basing on the cell viability, we choose the PHG concentration of 100 *μ*M and 10 mM concentration of H_2_O_2_ for further experiments (Figure 1).

### 2.2. Antioxidant Defense

Antioxidants are substances that, in low concentrations, protect against oxidation or delay the oxidation of cell components. In our study, we used both enzymatic (catalase, CAT; glutathione peroxidase, GSH-Px; glutathione reductase, GR; superoxide dismutase, SOD) and nonenzymatic (reduced glutathione, GSH) antioxidants to assess the antioxidant barrier.

#### 2.2.1. NAFLD Model

**Figure 2:**
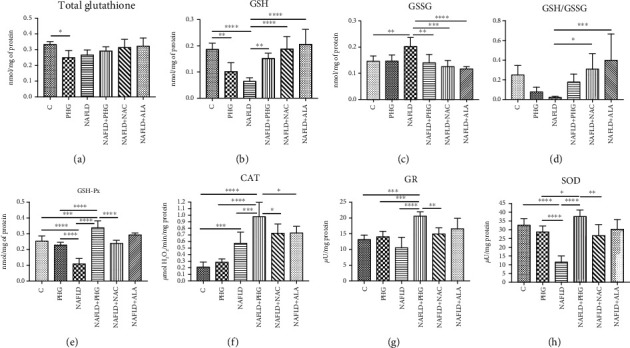
Antioxidant defense in the NAFLD model. The activity of the enzymatic and nonenzymatic antioxidants in HepG2 cell cultures incubated with phloroglucinol (PHG), palmitate (NAFLD), both PHG and NAFLD, and palmitate with other known antioxidants: N-acetylcysteine (NAFLD+NAC) and alpha-lipoic acid (NAFLD+ALA). (a) Phloroglucinol alone decreased the concentration of total glutathione; however, no effect was observed in the group incubated with palmitate and PHG together. (b–d) PHG similarly to other antioxidants normalised the level of reduced and oxidized glutathione but not the GSH/GSSG ratio. (e) The intensity of glutathione peroxidase activity (GSH-Px) was markedly elevated in the NAFLD+PHG group. (f) The activity of catalase (CAT) was significantly higher in the NAFLD+PHG group compared to other groups. (g) PHG increased the activity of glutathione reductase (GR) in lipid overload state (NAFLD+PHG). (h) PHG exerted no effect on superoxide dismutase compared with the control; however, the difference was significant between the NAFLD, PHG, and NAFLD+PHG groups.

PHG lowered cellular total glutathione content (PHG: -25.1% *p* < 0.05) and GSH concentration (PHG: -45.4%; NAFLD: -65.7% *p* < 0.0001; NAFLD+PHG: -18.8%, NAFLD+NAC: +0.9%, NAFLD+ALA: +10.3%). All the analyzed antioxidants decreased PA-induced GSSG (NAFLD:+38.8% *p* < 0.01; NAFLD+PHG: -3.9% *p* < 0.01; NAFLD+NAC: -14% *p* < 0.001; NAFLD+ALA: -19.9% *p* < 0.0001), and some of them normalised the GSH/GSSG ratio (NAFLD+NAC: -23.6% *p* < 0.05; NAFLD+ALA: -59.3% *p* < 0.001). Moreover, the activity of GSH-Px was markedly elevated in the NAFLD+PHG-treated group while being significantly lower in the group treated with NAFLD alone (PHG:-10.3%; NAFLD: -57.2% *p* < 0.0001; NAFLD+PHG: +33.5% *p* < 0.001; NAFLD+NAC: -5.8%; NAFLD+ALA: +15.5%). The activity of CAT was increased in all but one (PHG) of the experimental groups (PHG: +35.1%; NAFLD: +171.7% *p* < 0.01; NAFLD+PHG: +364% *p* < 0.0001; NAFLD+NAC: +244.7% *p* < 0.0001; NAFLD+ALA: +246.4% *p* < 0.0001) when compared to the control and significantly higher in NAFLD+PHG when compared to other groups. Additionally, PHG together with NAFLD elevated GR activity compared to all groups except NAFLD+ALA (PHG: +6.9%; NAFLD: -19.9%; NAFLD+PHG: +57.1% *p* < 0.0001; NAFLD+NAC: +13.7%, NAFLD+ALA: +26.3%). PHG had no substantial impact on SOD activity (PHG: -12%; NAFLD: -64.5% *p* < 0.0001; NAFLD+PHG: +15.2%, NAFLD+NAC: -18.2%, NAFLD+ALA:-7.2%) (Figure 2).

#### 2.2.2. H_2_O_2_ Model

**Figure 3:**
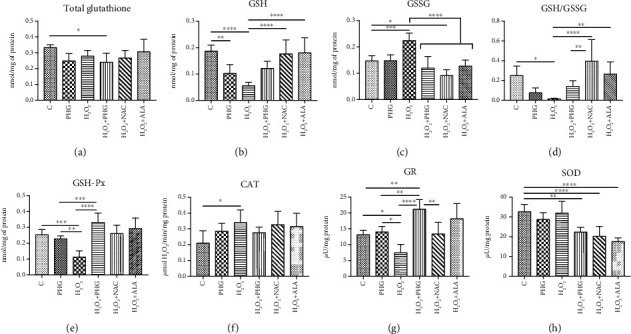
Antioxidant defense in the H_2_O_2_ model. The activity of the enzymatic and nonenzymatic antioxidants in HepG2 cell cultures incubated with phloroglucinol (PHG), hydrogen peroxide (H_2_O_2_), both PHG and H_2_O_2_, and hydrogen peroxide with other known antioxidants: N-acetylcysteine (H_2_O_2_+NAC) alpha-lipoic acid (H_2_O_2_+ALA). (a) The concentration of total glutathione (GSH) was slightly lowered only in the H_2_O_2_+PHG group. (b–d) PHG similar to other antioxidants normalised the level of reduced and oxidized glutathione but not the GSH/GSSG ratio. (e, f) Phloroglucinol significantly increased the activity of glutathione peroxidase (GSH-Px) decreased by H_2_O_2_. The exposure to H_2_O_2_ stimulated the activity of catalase (CAT). (g) The activity of glutathione reductase (GR) was intensified by phloroglucinol in the NAFLD+PHG group. (h) Phloroglucinol and other antioxidants decreased the activity of superoxide dismutase in NAFLD conditions.

Total glutathione was slightly decreased in the H_2_O_2_+PHG group (-27.8% *p* < 0.05). Both PHG and H_2_O_2_ alone depleted GSH with no significant differences to control in other groups (PHG: -45.3% *p* < 0.01; H_2_O_2_: -70% *p* < 0.0001) However, NAC and ALA normalised H_2_O_2_-induced GSH drop (H_2_O_2_+NAC: -5.5%; H_2_O_2_+ALA: -3.3% *p* < 0.0001). In contrast, H_2_O_2_ decreased GSH-Px activity with nonsignificant elevation in the group treated with H_2_O_2_+PHG (PHG: -10.3%; H_2_O_2_: -55.8% *p* < 0.001; H_2_O_2_+PHG: +30.7%; H_2_O_2_+NAC: +2.6%; H_2_O_2_+ALA: +15.4%). H_2_O_2_ significantly increased the GSSG concentration which was normalised by all analyzed antioxidants (H_2_O_2_: +52.8% *p* < 0.001; H_2_O_2_+PHG: -18.7%; H_2_O_2_+NAC: -37.9%; H_2_O_2_+ALA: -13.5% *p* < 0.0001). GSH/GSSG ratio was decreased by H_2_O_2_ and normalised by NAC and ALA (H_2_O_2_: -94.1% *p* < 0.05; H_2_O_2_+NAC: +58.3% *p* < 0.0001; H_2_O_2_+ALA: +6% *p* < 0.01). The H_2_O_2_+PHG group generated a rise of GR activity, in opposition to the group with H_2_O_2_ only, in which we noted low activity of GR (PHG:+6.9%; H_2_O_2_: -43.1% *p* < 0.05; H_2_O_2_+PHG: +61.7% *p* < 0.01; H_2_O_2_+NAC: +2.1%; H_2_O_2_+ALA: +38.7%). CAT activity was significantly increased only by incubation with H_2_O_2_ (PHG: +35.1%; H_2_O_2_: +61% *p* < 0.05; H_2_O_2_+PHG: +30.6%; H_2_O_2_+NAC: +54.5%; H_2_O_2_+ALA: +49.3%). PHG, NAC, and ALA together with H_2_O_2_ decreased the SOD level (PHG: -12%; H_2_O_2_: -2%; H_2_O_2_: +PHG: -31.4% *p* < 0.01; H_2_O_2_+NAC: -38% *p* < 0.0001; H_2_O_2_+ALA: -46.1% *p* < 0.0001) (Figure 3).

### 2.3. ROS Production and Nitrosative Stress

For the evaluation of ROS production rate, we determined the NADPH oxidase activity (NOX), which is the main prooxidative enzyme responsible for the formation of free radicals in the cell. For the assessment of nitrosative stress, we used both nitric oxide (NO) and peroxynitrite (the most reactive form of RNS (reactive nitrogen species)).

#### 2.3.1. NAFLD Model

**Figure 4:**
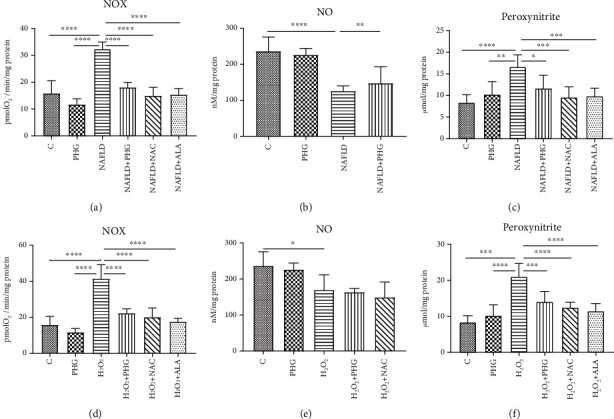
Concentration of the nitrosative stress products in HepG2. Cell cultures incubated with (first row) phloroglucinol (PHG), palmitate (NAFLD), both PHG and NAFLD, or palmitate with other known antioxidants: N-acetylcysteine (NAFLD+NAC) and alpha-lipoic acid (NA-FLD+ALA); (second row) phloroglucinol (PHG), hydrogen peroxide (H_2_O_2_), both PHG and H_2_O_2_, and hydrogen peroxide with other known antioxidants: N-acetylcysteine (H_2_O_2_+NAC) alpha-lipoic acid (H_2_O_2_+ALA). (a, c, d, f) The concentration of NADPH oxidase (NOX) and peroxynitrite was elevated significantly in NAFLD and H_2_O_2_ groups, respectively. (b, e) The concentration of nitric oxide was lower in the NAFLD and H_2_O_2_ groups.

Both NOX and peroxynitrite were markedly elevated in the NAFLD group (PHG: -26.2%; NAFLD: +103.6% *p* < 0.0001; NAFLD+PHG: +13.8%; NAFLD+NAC: -5.4%; NAFLD+ALA: -3.1%) (PHG: +22%; NAFLD: +98.3% *p* < 0.0001; NAFLD+PHG: +39.4%; NAFLD+NAC: +14.2%; NAFLD+ALA: +17.4%) as compared to the control. NO level was significantly lower in the NAFLD and NAFLD+PHG groups (PHG: -4.3%; NAFLD: -46.7% *p* < 0.0001; NAFLD+PHG: -37.5% *p* < 0.001) (Figure 4).

#### 2.3.2. H_2_O_2_ Model

Incubation with H_2_O_2_ resulted in substantially increased creation of peroxynitrite (PHG: -26.2%; H_2_O_2_: +161.2% *p* < 0.0001; H_2_O_2_+PHG: +40.7% *p* < 0.01; H_2_O_2_+NAC: +27%; H_2_O_2_+ALA: +10.5%) as well as elevated activity of NOX (PHG: +22%; H_2_O_2_: +150.6% *p* < 0.0001; H_2_O_2_+PHG: +67.8%; H_2_O_2_+NAC: +48.8%; H_2_O_2_+ALA: +36.7%). Concentration of NO was lowered in groups incubated with H_2_O_2_, H_2_O_2_+PHG, and H_2_O_2_+NAC (PHG: -4.3%; H_2_O_2_: -28.3% *p* < 0.05; H_2_O_2_+PHG: -30.8% *p* < 0.01; H_2_O_2_+NAC -36.8% *p* < 0.001) (Figure 4).

### 2.4. Protein Glycooxidative Damage

For the evaluation of protein glycooxidation products, we used oxidative modified amino acids (dityrosine, kynurenine, N-formylkynurenine, and tryptophan) and advanced oxidation protein products (AOPP), as well as beta-amyloid formation.

#### 2.4.1. NAFLD Model

**Figure 5:**
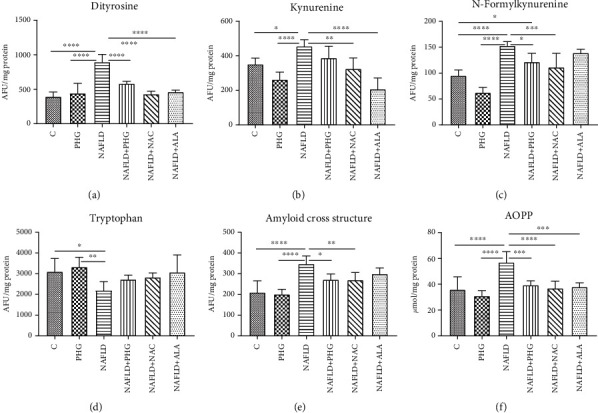
Protein glycooxidation damage in the NAFLD model. The concentration of the protein damage products in HepG2 cell cultures incubated with phloroglucinol (PHG), palmitate (NAFLD), both PHG and NAFLD, and palmitate with other known antioxidants: N-acetylcysteine (NAFLD+NAC) and alpha-lipoic acid (NAFLD+ALA). (a, b, e, f) The content of dityrosine, kynurenine, advanced oxidation protein products (AOPP), and the amyloid cross structure was increased significantly only in the NAFLD group. (c) The concentration of N-formylkynurenine was significantly elevated in the NAFLD group with a slight increase in the NAFLD+PHG group compared to the control (d). Concentration of tryptophan was decreased after incubation in NAFLD conditions compared with those of the control and PHG groups.

Incubation with PA resulted in significantly higher dityrosine (PHG: +12.2%; NAFLD: +126.5% *p* < 0.0001; NAFLD+PHG: +47.8% *p* < 0.01, NAFLD+NAC: +9%; NAFLD+ALA: +17.1%), kyneurenine (PHG: -25.3%; NAFLD: +29.8% *p* < 0.05; NAFLD+PHG: +10.5%, NAFLD+NAC: -7.6%; NAFLD+ALA: -40.9% *p* < 0.001), N-formylkynurenine (PHG: -34.2% *p* < 0.01; NAFLD: +60.8% *p* < 0.0001; NAFLD+PHG: +27.8% *p* < 0.05; NAFLD+NAC: +17.1%, NAFLD+ALA: +46.3% *p* < 0.001), AOPP (PHG: -13.7%; NAFLD: +58.6% *p* < 0.0001; NAFLD+PHG: +9.5%; NAFLD+NAC: +2.8%, NAFLD+ALA: +5.7%), and amyloid cross structure formation (PHG: -4.3%; NAFLD: +65.8% *p* < 0.0001; NAFLD+PHG: +279.5%; NAFLD+NAC: +28.6%, NAFLD+ALA: +42.5% *p* < 0.01). Additionally, formation of dityrosine and AOPP was significantly higher in the NAFLD group than in other groups. Tryptophan levels were found to be lower in the NAFLD-treated group (PHG: +7%; NAFLD: -29.4% *p* < 0.05; NAFLD+PHG: -12.2%; NAFLD+NAC: -8.8%, NAFLD+ALA: -1%) (Figure 5).

#### 2.4.2. H_2_O_2_ Model

**Figure 6:**
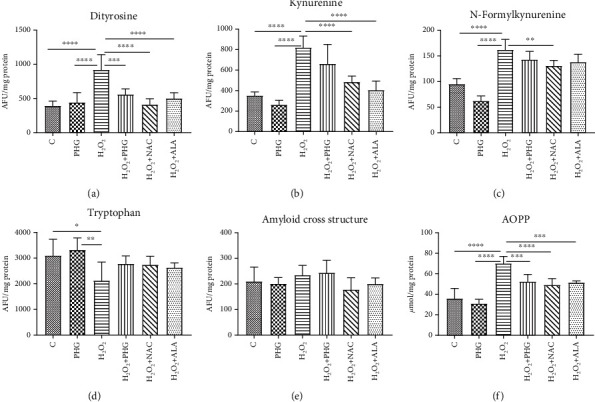
Protein glycooxidation damage in the H_2_O_2_ model. The concentration of the protein damage products in HepG2 cell cultures incubated with phloroglucinol (PHG), hydrogen peroxide (H_2_O_2_), both PHG and H_2_O_2_, and hydrogen peroxide with other known antioxidants: N-acetylcysteine (H_2_O_2_+NAC) alpha-lipoic acid (H_2_O_2_+ALA). (a–c, f) The content of dityrosine, kynurenine, N-formylkynurenine, and advanced oxidation protein products (AOPP) was raised in the H_2_O_2_ group only. (d) The content of tryptophan was significantly lower in the H_2_O_2_ group compared to the control and PHG. (e) No differences in the concentration of amyloid cross structure between groups were observed.

Levels of dityrosine (PHG: +12.2%; H_2_O_2_: +133.2% *p* < 0.0001; H_2_O_2_+PHG: +42.4%; H_2_O_2_+NAC: +4.7%; H_2_O_2_+ALA: +27.1%) and AOPP (PHG: -13.7%; H_2_O_2_: +95.4% *p* < 0.0001; H_2_O_2_+PHG: +45.8% *p* < 0.01; H_2_O_2_+NAC: +37% *p* < 0.05; H_2_O_2_+ALA: +43.6% *p* < 0.01) were substantially elevated in the group exposed to H_2_O_2_. By comparison, concentration of tryptophan in the same group was moderately but significantly lower compared to that in the control (PHG: +7%; H_2_O_2_: -31.4% *p* < 0.05; H_2_O_2_+PHG: -10.5%; H_2_O_2_+NAC: -11.6%; H_2_O_2_+ALA: -15.2%). N-formylkynurenine level was decreased in the PHG group and significantly elevated in other examined groups (PHG: -34.2% *p* < 0.01; H_2_O_2_: +70.4% *p* < 0.0001; H_2_O_2_+PHG: +50.4% *p* < 0.0001; H_2_O_2_+NAC: +37.9% *p* < 0.01; H_2_O_2_+ALA: +45.5% *p* < 0.001). There was no effect on the amyloid cross structure observed (PHG: -4.3%; H_2_O_2_: +11.8%; H_2_O_2_+PHG: +16.1%; H_2_O_2_+NAC: -15.2%; H_2_O_2_+ALA: -4.7%) (Figure 6).

### 2.5. Lipid and Carbonyl Damage

We assessed the oxidative damage to lipids by evaluating the concentration of malondialdehyde (MDA) and lipid hydroperoxides (LOOH). To assess carbonyl stress, we used advanced glycation end products (AGE).

#### 2.5.1. NAFLD Model

**Figure 7:**
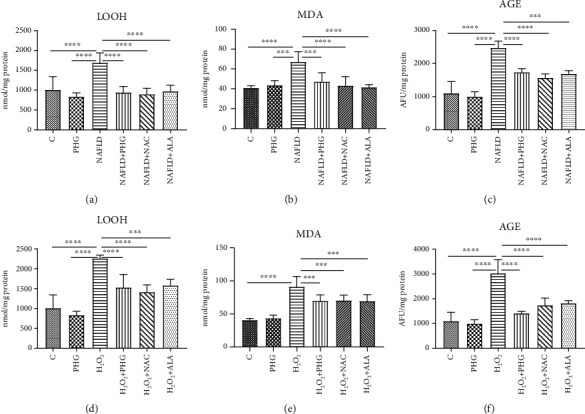
Lipid and carbonyl damage. The concentration of the lipid and carbonyl damage products in HepG2 cell cultures incubated with (first row) phloroglucinol (PHG), palmitate (NAFLD), both PHG and NAFLD, and palmitate with other known antioxidants: N-acetylcysteine (NAFLD+NAC) and alpha-lipoic acid (NAFLD+ALA); (second row) phloroglucinol (PHG), hydrogen peroxide (H_2_O_2_), both PHG and H_2_O_2_, and hydrogen peroxide with other known antioxidants: N-acetylcysteine (H_2_O_2_+NAC) alpha-lipoic acid (H_2_O_2_+ALA). (a–f) Concentration of total hydroperoxides (LOOH), malondialdehyde (MDA), and advanced glycation end products (AGE) was markedly elevated only in groups incubated in NAFLD and H_2_O_2_ conditions alone.

It was revealed that LOOH and MDA levels were substantially increased in the group exposed to PA (PHG: -17.6%; NAFLD: +66.8% *p* < 0.0001; NAFLD+PHG: -6.7%, NAFLD+NAC: -11.3%, NAFLD+ALA: -3.8%) (PHG: +6.5%; NAFLD: +64.7% *p* < 0.0001; NAFLD+PHG: +15.7%, NAFLD+NAC: +5.7%, NAFLD+ALA:+1.7%) whereas AGE levels rose in NAFLD, NAFLD+PHG, NAFLD+NAC, and NAFLD+ALA groups (PHG: -1.2%; NAFLD: +144.8% *p* < 0.0001; NAFLD+PHG: +71.7% *p* < 0.0001; NAFLD+NAC: +55.3% *p* < 0.01; NAFLD+ALA: +67.2% *p* < 0.001) as compared to the control (Figure 7).

#### 2.5.2. H_2_O_2_ Model

In comparison to the control, LOOH and MDA levels were increased significantly in the group treated with H_2_O_2_, H_2_O_2_+PHG, H_2_O_2_+NAC, and H_2_O_2_+ALA (PHG: -17.6%; H_2_O_2_: +123.2% *p* < 0.0001; H_2_O_2_+PHG: +51.4% *p* < 0.01; H_2_O_2_+NAC: +39.7% *p* < 0.05; H_2_O_2_+ALA: +56.2% *p* < 0.01) (PHG: +6.5%; H_2_O_2_: +122.8% *p* < 0.0001; H_2_O_2_+PHG: +71% *p* < 0.0001; H_2_O_2_+NAC: +72.5% *p* < 0.0001; H_2_O_2_+ALA: +69.9% *p* < 0.001) while significant growth in AGE levels was registered in H_2_O_2_, H_2_O_2_+NAC, and H_2_O_2_+ALA sets (PHG: -1.2%; H_2_O_2_: +199.1% *p* < 0.0001; H_2_O_2_+PHG: +39.8%; H_2_O_2_+NAC: +71.7% *p* < 0.05; H_2_O_2_+ALA: +79.5% *p* < 0.01) (Figure 7).

### 2.6. Inflammation, Hypoxia, and Apoptosis

**Figure 8:**
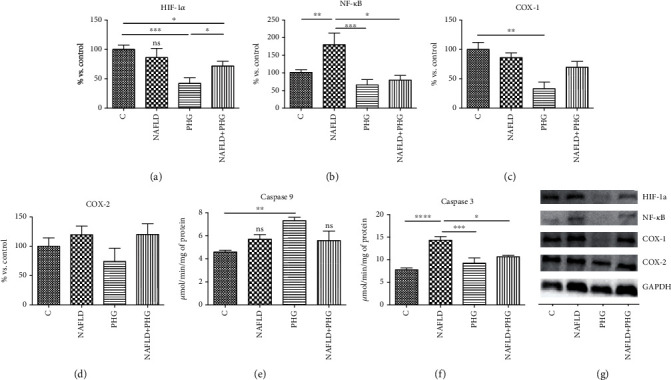
Inflammation, hypoxia, and apoptosis. PHG significantly reduced HIF-1*α* level (a) and PA induced NF-*κ*B activation (b) in HepG2 cells. Furthermore, it selectively inhibited COX-1 (c) with no effect on COX-2 (d). PHG stimulated Caspase 9 (e) and inhibited steatosis-induced activation of Caspase 3, showing its antiapoptotic properties (f). The expression of proteins involved in the inflammatory process assessed in Western blot (g). The band intensity was calculated as a % of control.

**Figure 9:**
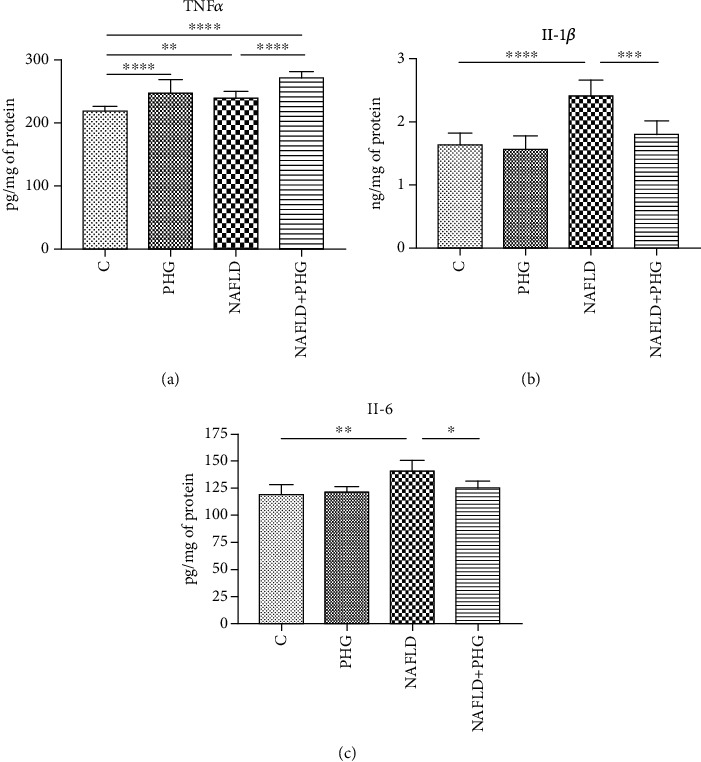
Proinflammatory cytokines. The TNF*α* concentration in the incubation media was slightly elevated in all the experimental groups when comparing to the control (a). Cell steatosis significantly increased the media level of main proinflammatory interleukins: Il-1*β* and Il-6 which were normalised when PHG was added (b, c).

The expression of proteins involved in the inflammatory and apoptotic pathways was assessed only in the main experimental and NAFLD model. PHG, both alone and combined with PA, decreased significantly the HIF-1*α* level compared to the control (PHG: -57.8%, PA+PHG: -28.3%; *p* < 0.05). What is more, we found that PA incubation increased NF-*κ*B expression (PA: +80%; *p* < 0.05), which was normalised when PHG was added to the steatotic medium (PA+PHG: -100.4%; *p* < 0.01). PHG also selectively inhibited COX-1 (PHG: -66.8%; *p* < 0.01) but did not significantly affect COX-2 (Figure 8). Furthermore, both PHG and PA increased TNF*α* levels in the incubation media (PA: +9.4%; PHG: +12.8%, PA+PHG: +24.2%; *p* < 0.05). PHG significantly lowered PA induced elevation of Il-1*β* (PA: +32.4%, *p* < 0.0001; PA+PHG: +10.3%, *p* < 0.001) and Il-6 (PA: +23.2%, *p* < 0.01; PA+PHG: +5.2%, *p* < 0.05) (Figure 9). It was revealed that PA activated Caspase 9 (PA: +59.4%; *p* < 0.01) and Caspase 3 (PA: +83.7%; *p* < 0.0001), which was normalised when PHG was added to the incubation media (PA+PHG: -25.5%; *p* < 0.05) (Figure 8).

## 3. Discussion

**Figure 10:**
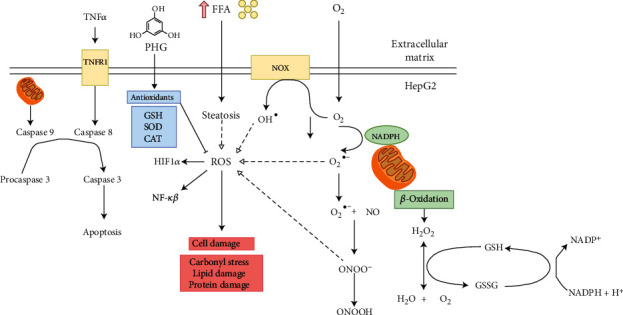
The potential mechanism underlying oxidative/nitrosative stress in nonalcoholic fatty liver disease (NAFLD).

This is the first study to assess the effect of PHG on redox homeostasis and oxidative/nitrosative damage in HepG2 cells treated with palmitic acid and hydrogen peroxide. We have shown that PHG strengthens the enzymatic and nonenzymatic antioxidant barrier and prevents oxidative/nitrosative stress comparable to other commonly used antioxidants. Additionally, PHG reduces inflammation and apoptosis in the NAFLD model (Figure 10).

NAFLD is one of the most underestimated diseases in the XXI century. Despite its severe aftermaths, such as T2DM, obesity, liver cirrhosis, and HCC, commonly occurring in the developed countries, it still does not have an acknowledged and efficient treatment method [26, 27]. What is more, NAFLD, as a root cause of liver cirrhosis and HCC, became one of the main reasons for liver transplantation in countries with western dietary pattern [28]. NAFLD's clinical significance in creating its long-term effects may be explained by the “two-hit” theory [29]. Following this theory, the first hit is related to excessive lipid accumulation in the liver, mostly due to an unbalanced diet and overnutrition, resulting in hepatocyte steatosis. The second hit is represented by the lipotoxicity of accumulated lipids, leading to increased oxidative stress, impaired metabolic function, inflammatory process, and NASH development [30]. Indeed, the critical role of redox imbalance in the pathogenesis of fibrosis and steatosis in NAFLD patients has recently been highlighted [31]. Interestingly, there is a significant overproduction of hepatic hydrogen peroxide and progressive depletion of glutathione reserves in NAFLD patients, leading to enhanced protein and lipid oxidative injury. That is why finding new approaches to fight NAFLD and introducing them into clinical practice seems to be crucial to prevent the development of liver steatosis aftermath. So far, numerous compounds, such as resveratrol, quercetin, enterolactone, vitamin E, ALA, or NAC, have been tested for that purpose [27, 32–36]. However, despite promising results *in vitro*, most of the polyphenols have very poor bioavailability in humans, and some have numerous side effects that limit their clinical usability. Thus, in our study, we focused on PHG, a phenolic antioxidant characterized by excellent pharmacokinetics, which may be found in seaweeds such as *Ecklonia cava* or *Cystoseira discors* [22, 37, 38]. PHG was discovered in 1855, yet it was not used in applications other than as an antispasmodic drug. It has a similar structure to resveratrol, and as we show herewith, similar to it, PHG decreases liver steatosis, strengthens antioxidative barriers, and reduces inflammation. In the following study, we assessed properties of PHG both in NAFLD and in hydrogen peroxide model and compared its effects with routinely used antioxidants—NAC and ALA.

In NAFLD, the excesses of saturated fatty acids, especially palmitic acid, accumulated in the liver inducing oxidative stress due to impaired mitochondrial *β*-oxidation and the generation of lipotoxic intermediates such as ceramides, diacylglycerols, and lysophosphatidylcholine [30]. A significant source of oxidative stress at NAFLD is the overproduction of hydrogen peroxide, so we decided on a second experimental model in our study. 50% growth inhibition (GI_50_) of H_2_O_2_ equal to around 10 mM was obtained in the MTT test. PHG was nontoxic to HepG2 cells in concentrations below 200 *μ*M. It reduced visible lipid accumulation and decreased the active form of Caspase 3 in the palmate-induced steatosis model (Figure 1). Since overproduction of ROS and reduced antioxidant defense are among the effects of excessive lipid accumulation in the liver, we analyzed the cellular content of enzymatic and nonenzymatic antioxidants [13, 14]. The exposure to both palmitate and H_2_O_2_ resulted in a decreased level of reduced glutathione (GSH), which is the major nonenzymatic antioxidant in the liver [14, 39]. The addition of antioxidants is accompanied by an increase of enzymes involved in the restoration of a reduced form of glutathione (GR, GSH-Px) and other enzymatic antioxidants such as SOD and CAT. These effects were observed in both experimental models with a slight predominance of the NAFLD model. Interestingly, within all analyzed compounds (NAC, ALA, and PHG), PHG had the most decisive influence on the enzymatic antioxidant barrier (Figures 2 and 3). Only the increase in GSH level and redox ratio was significantly lower compared to NAC and ALA. However, this should not come as a surprise because the latter are direct precursors to glutathione biosynthesis. This is particularly important because NAC and ALA have a proven therapeutic effect in NAFLD therapy. However, their limited use is due to the need to administer very high doses (up to 0.5 g per kg of body weight), causing numerous side effects [35, 40]. Although it is difficult to predict the side effects of PHG without human studies, we do not know any of the harmful actions of this compound. As far as enzymatic and nonenzymatic antioxidants are concerned, our observations are consistent with Quéguineur et al., who analyzed the dose-dependent effects of PHG on tert-butyl hydroperoxide-induced oxidative stress [41]. Despite the differences in used concentrations and experimental models, this seems to confirm the beneficial role of PHG in strengthening the liver antioxidant defense. On the other hand, PHG, in contrast to myricetin and pyrogallol, failed to increase antioxidant defense in yeast *Saccharomyces cerevisiae* what might suggest an animal-specific effect of PHG [42]. Although we did not assess the rate of hydrogen peroxide production, probably, the observed increase in GSH-Px and CAT activity does not result from the adaptive reaction to the increased formation of H_2_O_2_ by phloroglucinol. Previous studies showed that PHG elevates CAT activity and its protein expression, while CAT inhibitor abolished the protective effect of PHG from H_2_O_2_-induced cellular damage [43].

In animals, the primary source of ROS generation in physiological conditions is *β*-oxidation. However, in NAFLD, lipotoxic intermediates created by the incomplete oxidation of acyl-carnitine may also constitute an additional source of ROS [14]. Liver steatosis and exposure to H_2_O_2_ may also activate membrane NADPH oxidase (NOX), which catalase the process of superoxide anion formation [44]. Under these conditions, NO is also synthesized by inducible NO synthase (iNOS). iNOS is present in the liver and may be upregulated due to steatosis, cirrhosis, and liver cholestasis [45, 46]. Interestingly, the interaction of superoxide anions with NO results in highly reactive peroxynitrite, which might explain lowered NO concentration in experimental models. Indeed, peroxynitrite is one of the strongest prooxidizing factors in living organisms [47, 48]. Peroxynitrite and its derivatives react both with amino acids (including tyrosine, cysteine, and tryptophan), lipids, and several antioxidants. Peroxynitrite causes the formation of carbonyl groups, dimerization, nitration, and nitrosylation of amino acids and thiol compounds [48–50]. It was shown that nitrosative stress plays a critical role in various pathological conditions such as cardiovascular diseases, liver cirrhosis, diabetes, or cancer [22, 51]. In our experiment, we observed that all analyzed antioxidants presented a similar ability to diminish nitrosative stress both in the NAFLD and H_2_O_2_ models (Figure 4).

Once we established the antioxidant status and the level of the primary oxidative stress sources, we wanted to verify if PHG could prevent the formation of oxidative damage products. One of the most commonly used markers of overall protein damage is AOPP, which is created in interaction with chlorinated oxidants [52]. AOPP, similarly to MDA, CAT, and serum lipids, have been used recently to create a multimarker test aimed at improving the early identification of NAFLD and prediabetic patients [53]. As depicted in Figures 5 and 6, all analyzed compounds were efficient in decreasing AOPP. The increase of AOPP in experimental models can correspond with the lowered cellular GSH level (Figures 2 and 3) which suggests that protein oxidation in the liver is a result of a diminished antioxidant barrier [54]. Other commonly used markers of ROS generated protein damage are oxidized forms of tryptophan: N-formylkynurenine and kynurenine. N-formylkynurenine results from posttranslational oxidation of tryptophan, which may be further converted into kynurenine [55]. ALA was the most potent among tested antioxidants in diminishing kynurenine, while PHG and NAC were more efficient in lowering the level of N-formylkynurenine (Figures 5 and 6). Furthermore, we evaluated the level of dityrosine; a ROS-modified amino acid responsible for amyloid cross-linking, and the generation of A*β* plaques [56]. Both exposures to palmitate and H_2_O_2_ resulted in a significant increase of dityrosine, which was normalised in the presence of antioxidants. Interestingly, elevated amyloid cross structure content was observed only in the NAFLD model and partly decreased due to PHG and NAC action (Figures 5 and 6). Finally, we assessed the lipid and carbonyl damage products. Similar to proteins, both NAFLD and H_2_O_2_ caused a steep increase of oxidized forms of lipids such as MDA or LOOH and the elevation of glycation end products that were efficiently lowered when PHG, NAC, or ALA were added to the incubation media (Figure 7). Reducing the oxidation/nitrosylation of liver proteins and lipids may slow down the progression of NAFLD. It is well known that the increase in protein and lipid glycation is responsible for the development of ischemia and hepatic fibrosis, and thus, the progression of NAFL to NASH [14–57]. The accumulation of AGE and AOPP in the liver not only will increase the production of ROS (through NOX induction in the positive feedback mechanism) but also increases the expression of Fas ligand (protein from tumor necrosis factor (TNF) family) and activates the NF-*κ*B transcription factor (nuclear factor kappa-light-chain-enhancer of activated B cells), which stimulates neutrophil chemotaxis in the hepatocytes [31, 58] In our study, PHG, by strengthening the antioxidant barrier, not only reduces ROS production and oxidative/glycooxidation damage to proteins and lipids but also prevents excessive nitrosylation of the cell.

To assess the influence of PHG on inflammation and cell survival, we analyzed the expression of key proteins involved in apoptosis. As depicted in Figures 1 and 8, PHG revealed antiapoptotic properties by inhibiting the caspase cascade in the steatotic liver. The drop in Caspase 3 activity corresponded to lowered HIF-1*α* which might explain increased HepG2 survival. HIF-1*α* upregulates FOXO3, which is responsible for promoting Bax over Bcl-2 signaling [59]. In contrast to antiapoptotic Bcl-2, Bax opens voltage-dependent anion channels and creates pores in the mitochondrial outer membrane that initiate apoptosis [60]. Another interesting observation was a PHG-induced downregulation of NF-*κ*B, a key regulator of proinflammatory signaling (Figure 8). As a result of its activation, NF-*κ*B raises the TNF*α* level and stimulates iNOS, and COX-2 leads to proinflammatory prostaglandins and NO [61]. Despite the lack of statistically significant changes in COX-2, PHG inhibited COX-1 which may suggest its selectivity towards this isozyme (Figure 8). Nevertheless, the increased NO synthesis and inflammation results in oxidative/nitrosative stress and the creation of ROS and RNS, which weakens the antioxidative defense, and results in elevated protein accumulation, lipid, and carbonyl damage products. Finally, NF-*κ*B may also induce HIF-1*α* expression as a downstream effect of the PI3K-Akt-NF-*κ*B signaling pathway [62].

Our study confirms previous reports on the antioxidant properties of PHG. Indeed, it was shown that PHG protected human HaCaT keratinocytes against ultraviolet B- (UVB-) induced oxidative stress by scavenging intracellular ROS production [63]. PHG also decreased serum glucose level and formation of AGE in streptozotocin-induced diabetic rats [64]. Reduced oxidative stress under the influence of PHG was also noted in endothelial, neuronal, retinal, and neoplastic cells, which indicates the possibility of PHG as a promising therapeutic agent in several diseases [65–68]. A good pharmacokinetic profile and few PHG side effects are the additional advantages of this compound. However, as the mechanism of the antioxidant/antiglycation effect of PHG is not yet exactly known, further research is needed.

Nevertheless, our manuscript also has some limitations. We have evaluated the effect of PHG only on HepG2 cells, so further studies on other cell lines are necessary. Furthermore, we have only assessed selected oxidative/nitrosative stress biomarkers, so we cannot fully characterize the effect of PHG on NAFLD redox homeostasis. The next step is also to evaluate the therapeutic effect of PHG on the animal model and choose the dose characterizing the maximum therapeutic effect.

## 4. Conclusions

To sum up, our study showed that NAFLD and hydrogen peroxide models are comparable and suitable for assessing the oxidative/nitrosative stress in the liver. Although our study does not fully explain the PHG action's mechanism, this compound may be considered a new nutraceutical in counteracting NAFLD and preventing its severe molecular and clinical aftermath. Its effectiveness is comparable with other renowned antioxidants *α*-lipoic acid and N-acetylcysteine, which brings a promising perspective for the therapeutical application of phloroglucinol. The potential mechanism underlying oxidative/nitrosative stress in NAFLD are depicted on Figure 10.

## 5. Materials and Methods

### 5.1. Cell Culture

The study was conducted on HepG2 cells obtained from ATCC (American Type Culture Collection). The cells were incubated in DMEM (Dulbecco's modified Eagle's medium) enriched with 10% fetal bovine serum (FBS) and 1% penicillin/streptomycin for five days at 37°C in a humidified atmosphere containing 5% of CO_2_ until they will reach a confluence of 70%. The media were changed every 48 h preceded by rinsing in PBS. Subsequently, cells were transferred to 6-well plates and cultured in the growth medium until they achieved 90% of confluence. Then experimental incubations were conducted. Subsequently, cells were scrubbed in ice-cold RIPA buffer containing protease and phosphatase inhibitors (Roche Diagnostics GmbH, Germany) and ultrasonicated (Hielscher UP50H, Germany).

### 5.2. Experimental Models

To induce steatosis, cells were serum-starved for five h in a medium deprived of glucose and then incubated for 16 h in media containing either 0.75 mM palmitate alone or both PA and experimental compounds: 100 *μ*M PHG or 100 *μ*M ALA or 10 *μ*M NAC. Sodium palmitate was dissolved in absolute ethanol and heated to 70°C before conjugation with 10% fatty acid-free bovine serum albumin (BSA). Subsequently, the palmitic acid solution was added to serum-free DMEM supplemented with 10 mM Hepes, similarly to previously described methods [26, 27, 69]. The H_2_O_2_ model was prepared simply by adding an appropriate amount of hydrogen peroxide to the standard growth medium. Sigma-Aldrich, Poland, provided all the compounds.

### 5.3. Redox Homeostasis

The performed analyses included determination of antioxidant enzymes [catalase (CAT), glutathione peroxidase (GSH-Px), glutathione reductase (GR), and superoxide dismutase (SOD)] and nonenzymatic antioxidants [GSH], determination of prooxidant enzymes (NADPH oxidase, NOX), determination of oxidative damage to proteins [advanced glycation end products (AGE) and advanced oxidation protein products (AOPP)] and lipids [malondialdehyde (MDA) and total lipid hydroperoxides (LOOH)], and determination of protein glycooxidative products [dityrosine, kynurenine, N-formylkynurenine, and tryptophan], as well as the determination of nitrosative stress products [nitric oxide (NO) and peroxynitrite]. The absorbance/fluorescence was analyzed using the Infinite M200 PRO Microplate Reader (Tecan, Männedorf, Switzerland). All results were standardized to mg of the total protein. In the analysis of redox homeostasis and oxidative damage products, we followed the methods of Maciejczyk et al. [70].

### 5.4. Enzymatic and Nonenzymatic Antioxidants

The activity of catalase (CAT, EC 1.11.1.6) was estimated using the colorimetric method by measuring hydrogen peroxide (H_2_O_2_) decomposition at 240 nm [71, 72]. One unit of CAT activity was defined as the quantity of the enzyme catalyzing decomposition of 1 mmol H_2_O_2_ per 1 min. The activity of glutathione peroxidase (GSH-Px, EC 1.11.1.9) was analyzed colorimetrically by measuring the NADPH oxidation at 340 nm [73]. One unit of GPx activity was defined as the quantity of enzyme catalyzing the oxidation of 1 mmol NADPH per 1 min. The activity of glutathione reductase (GR, EC 1.8.1.7) was analyzed colorimetrically by measuring the decrease in NADPH absorbance at 340 nm [74]. One unit of GR activity was defined as the amount of enzyme catalyzing the oxidation of 1 *μ*mol NADPH per 1 min. The activity of superoxide dismutase (SOD, EC 1.15.1.1) was determined colorimetrically by measuring the inhibition of adrenaline oxidation at 480 nm [71]. One unit of SOD activity was defined as the quantity of enzyme inhibiting adrenaline oxidation by 50%.

The level of total glutathione was measured based on an enzymatic reaction with 5,5′-dithiobis-(2-nitrobenzoic acid) (DTNB), NADPH, and GR [75]. Oxidized glutathione (disulfide glutathione, GSSG) was determined similarly to the assay performed for total glutathione. However, prior to the determination, the samples had been thawed and neutralized to pH 6–7 using 1 M chlorhydrol triethanolamine. Then, samples were incubated with 2-vinylpyridine. The level of reduced glutathione (GSH) was calculated from the difference between the level of total glutathione and disulfide glutathione [75]. Redox ratio was calculated using the formula [GSH]^2^/[GSSG] [76].

### 5.5. ROS Production and Nitrosative Stress

NADPH oxidase activity (NOX, EC 1.6.3.1) was analyzed by the luminescence method using lucigenin as an electron acceptor [77, 78]. One unit of NOX activity was defined as the quantity of enzyme required to release 1 nmol of the superoxide anion per 1 min. The cells used for ROS production measurements were cultured in pyruvate and antibiotic-free media.

The concentration of nitric oxide (NO) was determined using the Griess method based on the reaction of nitrates with sulfanilamide and N-(1-naphthyl)-ethylenediamine dihydrochloride [79, 80]. The absorbance was measured at 490 nm. The concentration of peroxynitrite was estimated colorimetrically based on peroxynitrite-mediated nitration of phenol to nitrophenol [81]. The absorbance was measured at 320 nm.

### 5.6. Protein Glycooxidation Products

The content of protein glycooxidation products (dityrosine, kynurenine, N-formylkynurenine, and tryptophan) was estimated fluorimetrically by measuring fluorescence at 330/415 nm (dityrosine), 365/480 nm (kynurenine), 325/434 nm (N-formylkynurenine), and 95/340 nm (tryptophan). Immediately before the assay, cells were diluted in 0.1 M H_2_SO_4_ (1 : 10, *v*/*v*). The results were normalised to fluorescence of 0.1 mg/mL quinine sulfate in 0.1 M H2SO4 [54]. The concentration of advanced oxidation protein products (AOPP) was estimated colorimetrically by measuring the sample's iodide ion oxidizing capacity at 340 nm [10].

### 5.7. Lipid and Carbonyl Damage

The concentration of total hydroperoxides (LOOH) was determined colorimetrically based on the reaction of Fe^3+^ (resulting from Fe^2+^ after its oxidation by LOOH) with xylenol orange [82]. The absorbance of the resulting complex was measured at 560 nm. Immediately before the assay, cells were diluted in 0.02 M PBS, pH 7.4 (1 : 5, *v* : *v*) [71]. The concentration of malondialdehyde (MDA) was determined colorimetrically using the thiobarbituric acid reactive substances (TBARS) method. The absorbance was measured at 535 nm, and 1,3,3,3-tetraethoxypropane was used as a standard [83]. The concentration of advanced glycation end products (AGE) was detected fluorimetrically by measuring AGE-specific fluorescence at 350/440 nm [84]. Immediately before the assay, cells were diluted in 0.02 M phosphate-buffered saline (PBS) pH 7.4 (1 : 5, *v* : *v*) [83].

### 5.8. In Situ Immunofluorescence

Approximately 10^6^ cells were seeded on a 12-well plate and grown overnight. The next day, cells were treated in the same manner as cells from the main part of the experiment. At the end of 16 h, experimental incubation cells were rinsed with PBS and fixed in 3.7% paraformaldehyde for 15 min, permeabilized with 0.1% Triton X-100 for 20 min, and blocked with 2% FBS 1% BSA in PBS for 30 min at room temperature. Subsequently, cells were incubated with the primary monoclonal anticleaved Caspase 3 antibody (1 : 200, Abcam, UK) and then with the secondary goat-anti-rabbit antibody conjugated with Alexa Fluor 488 (1 : 1000, Abcam, UK) both for 1 h at room temperature. Finally, cell nuclei were counterstained with DAPI (1 : 5000, Sigma-Aldrich). The images were obtained with a fluorescent microscope (Leica DMi8, Germany).

### 5.9. Oil Red O Staining

Approximately 10^6^ cells were seeded on a 12-well plate and grown overnight. The next day, cells were treated in the same manner as cells from the main part of the experiment. After experimental treatment, cells were fixed in 3.7% paraformaldehyde for 15 min and then stained with 0.5% Oil Red O solution. The images were obtained with a fluorescent microscope (Leica DMi8, Germany).

### 5.10. Western Blotting

Proteins of interest expression were analyzed using the standard Western blot technique. To standardize samples, total protein concentration was assessed using the bicinchoninic acid method (BCA) with BSA as a standard. Cell lysates were separated by 10% sodium dodecyl sulfate-polyacrylamide gel electrophoresis (SDS-PAGE) and transferred to nitrocellulose membranes. Subsequently, they were blocked with 5% nonfat dry milk and immunoblotted with primary antibodies of interest and incubated with secondary antibodies labeled with horseradish peroxidase (HRP). The protein bands were quantified densitometrically using the ChemiDoc visualisation system (Bio-Rad, Poland). Equal protein loading was controlled by Ponceau S staining. All the proteins' expression was standardized to the GAPDH (Santa Cruz Biotechnology, USA) expression, and the control was set as 100%.

### 5.11. ELISA

TNF*α*, IL-1, and IL-6 concentrations were analyzed using a standard ELISA kit purchased from Abcam, UK (TNF*α*) and EIAab, China (IL-1 and IL-6). All the procedures were made on cell culture media samples standardized to protein concentration following the manufacturer's instructions. The assay was done in triplicate, and the results were averaged.

### 5.12. Statistical Analysis

The results were expressed as mean ± SD based on six independent repetitions. Statistical significance was tested with one-way analyses of variance (ANOVA) and Tukey HSD post hoc test using GraphPad Prism 7 (GraphPad Software Inc., La Jolla, CA, USA). Multiplicity adjusted *p* value was also calculated. Results were considered statistically significant at *p* ≤ 0.05.

## Data Availability

The data used to support the findings of this study are available from the corresponding author upon request.
